# Childhood maltreatment and suicide attempts in prisoners: a systematic meta-analytic review

**DOI:** 10.1017/S0033291719002848

**Published:** 2019-10-30

**Authors:** Ioannis Angelakis, Jennifer L. Austin, Patricia Gooding

**Affiliations:** 1 University of South Wales, School of Psychology, Pontypridd, Wales, UK; 2 Division of Psychology and Mental Health, School of Health Sciences, Faculty of Biological, Medical and Health Sciences, University of Manchester, Manchester, UK; 3 Manchester Academic Health Science Centre, MAHSC, Manchester, UK

**Keywords:** Abuse, childhood maltreatment, meta-analysis, neglect, prisoners, suicide attempts, systematic review

## Abstract

In the past decade, the links between core types of childhood maltreatment and suicidal acts have become an increasingly important area of investigation. However, no meta-analytic review has examined this relationship in prisoners. We undertook the first systematic meta-analytic review examining the link between childhood maltreatment and suicide attempts in prisoners to redress this important gap. We searched Medline, PsychINFO, Embase, Web of Science and CINAHL from inception until August 2019. Meta-analyses using random effect models were applied, and heterogeneity was quantified using the *I*^2^ statistic. Publication bias and risk of bias across studies were assessed. We identified 24 studies comprising 16 586 prisoners. The rates of different types of childhood maltreatment ranged between 29% and 68% [95% confidence interval (CI) 18–81%]. The rate of suicide attempts in prisoners was 23% (95% CI 18–27%). Main results demonstrated that sexual abuse [odds ratio (OR) 2.68, 95% CI 1.86–3.86], physical abuse (OR 2.16, 95% CI 1.60–2.91), emotional abuse (OR 2.70, 95% CI 1.92–3.79), emotional neglect (OR 2.29, 95% CI 1.69–3.10), physical neglect (OR 1.57, 95% CI 1.27–1.94) and combined abuse (OR 3.09, 95% CI 2.14–4.45) were strongly associated with suicide attempts in prisoners. There was an indication of publication bias. Duval and Tweedie's trim-and-fill method was applied, which increased the odds for suicide attempts. Given the high rates of prison suicide deaths and suicide attempts, our findings suggest an urgent need for targeted suicide prevention priorities for prisoners, with a particular focus on ameliorating the effects of childhood traumatic experiences on suicidal prisoners.

Suicide fatalities are a major problem facing prisons, with male and female prisoners dying by suicide at a rate that is three- to nine-times higher than that found in the general population (Fazel *et al*., 2011, 2017). Furthermore, Fazel *et al*. (2017) reported that such fatalities have been elevated for prisoners in England and Wales, where suicide death rates were reported to be between 6 and 20 times higher than those recorded in the community. With regard to the geographical distribution of suicide deaths, Nordic and Western European countries have reported the highest suicide death rates, with over 100 suicide fatalities per 100 000 prisoners being recorded. In Australasian and North American countries, prison suicide death rates ranged between 23 and 67 per 100 000.

Given the high rates of prison suicide deaths globally, it is imperative to identify key factors that may contribute to suicidal acts in prisoners. Engaging in suicide attempts is a strong predictor of suicide deaths (Joiner, 2005; Johnson *et al*., 2010). Estimates of the prevalence of suicide attempts while in prison and those of the prevalence of attempts of current prisoners prior to being incarcerated have been reported as ranging between 10% and 31%, and between 10% and 54%, respectively (Godet-Mardirossian *et al*., 2011; Bhatta *et al*., 2014; Moore *et al*., 2015; Power *et al*., 2016; Sánchez *et al*., 2019). Estimates of lifetime suicide attempts within the general population range between 0.3% and 3% (Nock *et al*., 2008; Borges *et al*., 2010). Thus, an in-depth examination of the key variables that are linked with suicide attempts are essential in reducing the elevated rates of prison suicide deaths.

Adverse childhood experiences have been robustly associated with an increased risk for suicide attempts (Brezo *et al*., 2008; Kim *et al*., 2013). For example, a meta-analytic review which examined this association in clinical and non-clinical populations demonstrated a strong link between key types of childhood maltreatment (i.e. sexual, physical and emotional abuse, and emotional neglect) and both suicide attempts and ideation (Angelakis *et al*., 2019). Notably, these results were unaffected by key methodological variations (e.g. screening tools and/or research designs employed) and individual-level characteristics (e.g. age and gender) across studies. This demonstrates the robustness of this relationship.

In the last decade, links between myriad forms of childhood abuse and suicidal experiences have begun to be explored in incarcerated populations (Bhatta *et al*., 2014; Boonmann *et al*., 2016). Previous research has established that prisoners reliably report more childhood traumatic events than those in the general community (Jordan *et al*., 1996) and that these experiences are strongly linked to suicidal acts (Angelakis *et al*., 2019). Furthermore, with prison suicide death rates being substantially higher than those in the general population, a systematic meta-analytic review examining the link between childhood maltreatment and suicide behaviors in prisoners is critical for guiding future research priorities and associated policy changes. Currently, there is no critical synthesis of evidence pertaining to the relationship between childhood abuse and suicidal acts within individuals in custody. Hence, we undertook the first systematic meta-analytic review evaluating this relationship. There were two key objectives:

(i) to quantitatively analyze the associations between core forms of childhood maltreatment and suicide attempts in prisoners;
(ii) to examine the degree to which any links between childhood abuse and suicide attempts also apply to suicidal ideation.

## Method

The Preferred Reporting Items for Systematic Reviews and Meta-Analyses (PRISMA; Moher *et al*., 2009) and the Meta-Analysis of Observational Studies in Epidemiology (MOOSE; Stroup *et al*., 2000) statements were employed to conduct and report the findings of this systematic review and meta-analysis. The MOOSE checklist is available in the online supplemental material (Appendix A).

### Eligibility criteria

Studies were included that:

(a) reported a quantitative outcome of the association between any type of childhood maltreatment (e.g. sexual, physical, emotional abuse; physical and/or emotional neglect) experienced before the age of 18 years and suicide attempts or any other mode of suicidal experiences, such as thoughts, plans, intentions or deaths;
(b) focused on both juvenile (under 18) and adult (18 and over) prisoners;
(c) employed a quantitative research design;
(d) were written in English and published in peer-reviewed academic journals.

Studies were excluded that:

(a) focused on adverse childhood experiences other than abuse and neglect (e.g. death of a significant other, experiences of being bullied);
(b) reported data on the link between abuse and neglect experienced in adulthood (e.g. domestic abuse) and suicidal behaviors;
(c) were reviews, position papers, theses, and/or reports.

### Search strategy and data sources

Five electronic bibliographic databases, including Medline, PsychInfo, Embase, Web of Science, and CINAHL, were searched with an end date of August 2019. The reference lists of the identified studies were also checked to locate eligible studies, and authors were contacted if additional information was needed. The search strategy included both text words and Medical Subjective Headings (MeSH) terms and combined three main blocks of key-terms: suicide (suicid*, self* harm*, suicid* correl*), child/sexual/physical/emotional abuse or neglect or maltreatment or adversities (child*, sex*, phys*, emoti* abuse, negl*, maltreat*, advers*) and prisoners (prison*, inmat*, offend*, jail*, custod*, imprison*, correction*, correctional facilit*, institut*, felon*, delinquen*, disciplin*, criminal*).

### Study selection

Study selection was conducted in two stages. In stage 1, the first two authors (IA and JA) independently examined the titles and abstracts of the papers. In stage 2, the same raters evaluated whether all the inclusion and exclusion criteria were met. Inter-rater reliability for the title/abstract and full-text screening process was excellent (*κ* = 0.91 and 0.93, respectively).

### Data extraction

An electronic data extraction sheet was devised and piloted in five randomly selected papers to extract descriptive data on key study characteristics, including number and age of participants, research design, method of recruitment, type of childhood maltreatment, mode of suicidal experiences and behaviors, and quantitative data [e.g. odds ratios (ORs) or equivalent measures] for the meta-analysis. Once the data sheet was deemed appropriate, data extraction on the remaining articles was completed by the first two authors (IA and JA). Inter-rater agreement was excellent (*κ* = 0.93).

### Risk of bias assessment

The methodological quality of the studies was critically evaluated using the criteria as adopted by the Centre for Reviews and Dissemination (CRD, 2010) guidance for undertaking reviews in health care. These assessed the research design (cross sectional = 0, prospective/experimental = 1), response rate (⩽70% or not reported = 0, ⩾70% = 1), screening tools for childhood maltreatment and for suicide experiences (self-report scale/not reported = 0, structured/semi-structured clinical interview = 1), and control for confounding/other factors in the reported analyses (no control/not reported = 0, controlled = 1). Those studies that were scored with at least four criteria out of five were given a high-quality rating; those with less than or equal to three criteria were given a low-quality rating. A binary critical appraisal item was also created based on these ratings (1 = low quality appraisal score; 2 = high quality appraisal score) which was entered as a moderator in the meta-regression analyses.

### Data analyses

The primary outcome was the association between different forms of childhood maltreatment and suicide attempts. More than half of the studies (*k* = 16) reported suicide attempts as dichotomous outcomes (number/proportions of prisoners with or without experiences of childhood maltreatment who engaged in suicide behavior in adulthood), whereas the remaining eight studies reported such outcomes in different formats (e.g. mean score of suicide attempts in participants with and without history of childhood maltreatment). ORs were selected as the preferred effect size. Continuous data were converted to ORs by utilizing a widely used formula (Borenstein *et al*., 2009). Most of the included studies (*n* = 18) contributed more than one relevant effect size. Therefore, we ensured that all the different types of childhood maltreatment were pooled separately to avoid double counting of studies in the same analysis.

All data were analyzed in Stata 15^®^. We used the *metan* command to calculate the pooled effect size of the link between the different types of childhood maltreatment and suicide attempts. A random effects model was utilized because substantial heterogeneity was anticipated, which was assessed with the *I*^2^ statistic (Higgins *et al*., 2003). Traditionally, values of 25%, 50% and 75% indicate low, moderate and high heterogeneity, respectively. Publication bias was examined with funnel plots and by applying the Egger et al.'s (1997) test in cases where the analyses were based on at least nine studies (Saveleva and Selinski, 2008). Duval and Tweedie's (2000) trim-and-fill method, which yields an estimate of the number of the missing studies, was used to provide an adjusted effect size estimate in case of publication bias. The prevalence rates of the various forms of childhood maltreatment and suicide attempts in prisoners were calculated by using the *metaprop* command (random effects model due to anticipated heterogeneity; Nyaga *et al*., 2014), by excluding controls for those studies which recruited a mixed sample of participants (Rivlin *et al*., 2013; Brewer-Smyth *et al*., 2014). Finally, the extent to which methodological variations across the studies affected the link between childhood maltreatment and suicide attempts in prisoners was examined by applying univariate meta-regression models using the *metareg* command (Harbord and Higgins, 2008). We conducted such analyses only for comparisons comprising eight or more studies (Thompson and Higgins, 2002). Overall, two continuous and four categorical moderators were examined including: participants' age; percentage of males; screening method for childhood maltreatment and suicide attempts (1 = self-report survey, 2 = interview); timeframe of suicide attempts (1 = lifetime, 2 = prison, 3 = both) and quality appraisal score (1 = low, 2 = high).

## Results

**Fig. 1.**
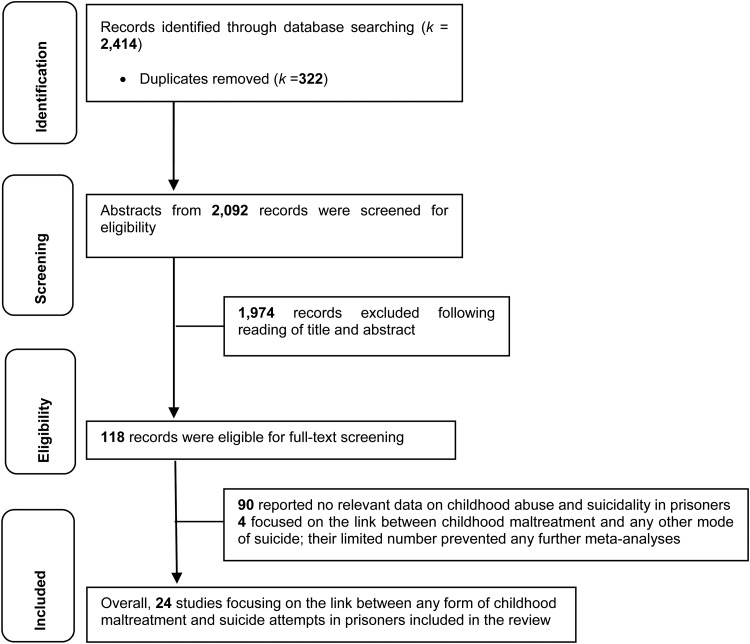
PRISMA 2009 flow diagram for the entire review.

We retrieved 2414 articles; 322 were removed as they were duplicates and a further 1974 were excluded because they (a) focused on other forms of adverse childhood experiences (e.g. parental death or divorce), (b) non-suicidal self-harm experiences (e.g. self-mutilation), (c) were non-empirical studies, or (d) were focused on misconduct, inappropriate behaviors, rule violation in prisons among others. The full texts of 118 articles were screened for inclusion. An additional 90 studies were excluded as they did not report data relevant to the link between childhood maltreatment and suicidal thoughts and behaviors. Of the remaining 28 studies, only four focused exclusively on either suicidal ideation (Zhang *et al*., 2010; Boonmann *et al*., 2016), suicide risk (Blaauw *et al*., 2002), or suicidality (a term that may incorporate attempts, ideation, plans, urges and intentions; Sergentanis *et al*., 2014), resulting in 24 independent studies which were focused exclusively on the link between any form of childhood maltreatment and suicide attempts in prisoners (see Fig. 1).

The overall sample was 16 586 prisoners across the 24 studies (see Table 1). The mean age was 32.39 (s.d. = 4.64, range: 17–41). Fifty-eight percent of participants were male. Most of the studies were conducted in the United States (*k* = 9; 37.5%), Italy (*k* = 4; 16.67%), or Australia (*k* = 3; 12.5%), whereas a single study (4.17%) was conducted in each of England, France, Spain, Sweden, Norway, Israel, Canada, and Turkey.

**Table 1.** Study characteristics

Study	Country	Study design	Screening tool for abuse	Mode of childhood abuse	Screening tool for suicidality	Mode of suicidality	Timeframe of suicidality	Nature of offence	Sample size *N*	Men (%)	Age (years)	Q.A.
Altinas and Bilici (2018)	Turkey	Cross-sectional	CTQ	All forms of abuse and neglect	CI	SA	Lifetime	n/r	216, response rate = 92.6%	50	Range: 18 and above	3/5
Bhatta *et al*. (2014)	United States	Cross-sectional	SRQ	Sexual abuse	SRQ	SA	Lifetime	Personal, property, drug, public order and unruly offences	3156, response rate = 100%	78.0	Range: 12–17	4/5
Brewer-Smyth *et al*. (2014)	United States	Case-control	MS	Physical and sexual abuse	n/r	SA	Current	Property and drug offences	113, response rate = 91.87%	0	n/r	2/3
Butler *et al*. (2001)	Australia	Cross-sectional	HSI	Sexual Abuse	HSI	SA	Lifetime	Violent and non-violent offenses	789, response rate = 90%	83.3	Median = 30, range: 18–77	4/5
Chapman *et al*. (2014)	United States	Cross-sectional	CTQ	All forms of abuse and neglect	LPC-II	SA	Lifetime/current	Personal, property, drug offenses	104, response rate = 88.89%	0	*M*_age_ = 31.94	3/5
Chen and Gueta (2017)	Israel	Cross-sectional	CTQ	All forms of abuse and neglect	ASI-I	SA	Lifetime	Drug and property offenses, prostitution and assault	46, response rate = 100%	0	*M*_age_ = 34.82	3/5
Clements-Nolle *et al*. (2009)	United States	Cross-sectional	CTQ	All forms of abuse and neglect	SBQ	SA	Lifetime	n/r	247, response rate = 54%	0	Median = 43, range: 20–72	3/5
DeCou *et al*. (2016)	United States	Cross-sectional	LHC	Sexual abuse	LHC	SA	Lifetime	n/r	491, response rate = 23.42%	0	*M*_age_ = 34, range: 17–55	2/5
Friestad *et al*. (2014)	Norway	Cross-sectional	CI	All forms of abuse and neglect	CI	SA	Lifetime	n/r	141, response rate = 75.4%	0	*M*_age_ = 33	4/5
Godet-Mardirossian *et al*. (2011)	France	Cross-sectional	CI	Combined forms of childhood maltreatment	MNI	SA	Current	n/r	899, response rate = 100%	100	Median = 37, range: 19–84	3/5
Gorodetsky *et al*. (2016)	Italy	Cross-sectional	CTQ	All forms of abuse and neglect	MNI	SA	Lifetime	n/r	702, response rate = 100%	100	n/r	3/5
Hakansson *et al*. (2011)	Sweden	Cross-sectional	ASI	Sexual, physical and emotional abuse	ASI	SA	Lifetime/current	n/r	1561, response rate = 84.93%	80.62	n/r	4/5
Jeglic *et al*. (2013)	United States	Cross-sectional	Third party information	Combined forms of childhood maltreatment	Third party information	SA	Lifetime/current	Sexual offenses	3030, response rate = 100%	100	*M*_age_ = 39.2, range: 13–85	3/5
Kenny *et al*. (2008)	Australia	Cross-sectional	CTQ	All forms of abuse and neglect	CI	SA	Lifetime/current	Violent crimes, property, drug, public order and other offenses	319, response rate = 76%	92	*M*_age_ = 16.55, range: 16–22	3/5
Mandelli *et al*. (2011)	Italy	Cross-sectional	CTQ	All forms of abuse and neglect	MNI	SA	Lifetime	Violent and non-violent offenses	2000, response rate = 77.6%	100	*M*_age_ = 40	4/5
Moore *et al*. (2015)	Australia	Cross-sectional	CTQ	All forms of abuse and neglect	SBQ	SA	Lifetime/current	n/r	361, response rate = 86.70%	87.54	n/r	3/5
Power *et al*. (2016)	Canada	Cross-sectional	CTQ	All forms of abuse and neglect	CI	SA	Lifetime	n/r	415, response rate = 100%	64.58	n/r	4/5
Rivlin *et al*. (2013)	England	Matched-case control	CTQ	All forms of abuse and neglect	Third party referrals from prison officers	SA	Current	Violent, sexual and property offenses	120, response rate = 100%	100	Range: 18 years and above	4/5
Roy *et al*. (2014)	Italy	Cross-sectional	CTQ	All forms of abuse and neglect	MNI	SA	Lifetime	Violent and non-violent offenses	1537, response rate = 100%	100	*M*_age_ = 39.6	3/5
Sánchez *et al*. (2019)	Spain	Cross-sectional	CTQ	Emotional physical abuse, and sexual abuse	A single question	SA	Current	Property offenses	1146, response rate = 82.29%	100	*M*_age_ = 36.9	1/5
Sarchiapone *et al*. (2009)	Italy	Cross-sectional	CTQ	All forms of abuse and neglect	MNI	SA	Lifetime	Violent and non-violent offenses	903, response rate = 100%	100	*M*_age_ = 40.74	3/5
Swogger *et al*. (2011)	United States	Cross-sectional	A single question	Physical abuse	A single question	SA	Lifetime	n/r	266, response rate = 100%	74.81	*M*_age_ = 33.7, range: 18–62	4/5
Tripodi *et al*. (2014)	United States	Cross-sectional	CTQ	All forms of abuse and neglect	ASI	SA	Lifetime	n/r	150, response rate = 83%	0	*M*_age_ = 34.3	4/5
Verona *et al*. (2005)	United States	Cross-sectional	CI	Physical and sexual abuse	CI	SA	Lifetime	n/r	226, response rate = 100%	0	*M*_age_ = 31.9	3/5

ASI, addiction severity index; ASI-I, Addiction Severity Index Interview; CI, Clinical Interview; CTQ, Child Trauma Questionnaire; HSI, Health Survey Interview; LHC, Life History Calendars; LPC-II, Lifetime Para-suicide Count-II; M, mean; MNI, Mini Neuropsychiatric Interview; MS, Muenzenmaier's Scale; n/r, not reported; Q.A, Quality appraisal of the methodology of the included studies; SA, suicide attempts; SBQ, suicidal behaviours; s.d., standard deviation; SRQ, Self-Report Questions

As detailed in Table 2, within the current sample, 29% of the prisoners reported being victims of sexual abuse [*k* = 11, 95% confidence interval (CI) 18–41%, *I*^2^ = 98.76%]; 43% had experienced physical abuse (*k* = 8, 95% CI 29–57%, *I*^2^ = 98.08%); 49% had experienced emotional abuse (*k* = 7, 95% CI 32–65%, *I*^2^ = 98.40%); and 68% had experienced emotional neglect (*k* = 3, 95% CI 53–81%, *I*^2^ = 86.16%).

**Table 2.** Rates of childhood maltreatment and suicide attempts in prisoners

	Prevalence			Heterogeneity
	*k*	%	95% CI	*I*^2^
Childhood maltreatment				
Sexual abuse	11	29	18–41	98.76%
Physical abuse	8	43	29–57	98.08%
Emotional abuse	7	49	32–65	98.40%
Emotional neglect	3	68	53–81	86.16%
Suicidality				
Suicide attempts	21	23	18–27	97.24%

*k*, number of independent prevalence estimates.

With regard to suicide attempts, 58.33% (*k* = 14) of the studies reported data on lifetime suicide attempts, which incorporated suicide attempts of prisoners while in prison and attempts prior to or after leaving the prison, 16.66% (*k* = 4) reported data on suicide attempts while in prison, and the remaining 20.83% (*k* = 5) focused on a combination of attempts that occurred either during or after imprisonment. Hence, provided that lifetime suicide attempts also incorporated those attempts that occurred exclusively in prisons, we pooled and reported the rates of suicide attempts into a single variable. The overall prevalence of the suicide attempts of prisoners was 23% (95% CI 18–27%, *I*^2^ = 97.24%) based on 21 studies (see Table 2; online Supplementary Appendix E).

### Main meta-analyses: associations between types of childhood maltreatment and suicide attempts in prisoners

All forms of childhood maltreatment were associated with significantly increased odds for suicide attempts in prisoners (see online Supplementary Appendix B for forest plots). Sexual and emotional abuse were associated with a three-fold increased likelihood for suicide attempts (*k* = 16, OR 2.68, 95% CI 1.86–3.86, *I*^2^ = 92.4% and *k* = 9, OR 2.70, 95% CI 1.92–3.79, *I*^2^ = 74.0%, respectively) whereas physical abuse and emotional neglect were associated with a two-fold increased likelihood for suicide attempts (*k* = 11, OR 2.16, 95% CI 1.60–2.91, *I*^2^ = 86.6% and *k* = 7, OR 2.29, 95% CI 1.69–3.10, *I*^2^ = 41.9%, respectively). Physical neglect was associated with 1.5-fold increased likelihood for suicide attempts (*k* = 7, OR 1.57, 95% CI 1.27–1.94, *I*^2^ = 89.2%). Almost half of the included studies provided data for the link between unspecified forms of childhood abuse/neglect and suicide attempts. This category was named ‘combined abuse’ and was analyzed separately. Combined abuse was associated with a three times increased likelihood for suicide attempts (*k* = 13, OR 3.09, 95% CI 2.14–4.45, *I*^2^ = 91.9%). As indicated by the *I*^2^ statistic, heterogeneity ranged from medium to high across all analyses, except for the association between emotional neglect and suicide attempts, where heterogeneity ranged from moderate to low.

### Publication bias

We assessed publication bias across comparisons that included at least nine studies. The screening of the funnel plots (see online Supplementary Appendix C) suggested that there was a publication bias for the relationship between sexual abuse and suicide attempts. Furthermore, the Egger's *et al*. (1997) test for publication bias was significant in all the comparisons examined, which was an indication of publication bias (see Table 3). Therefore, we ran the Duval and Tweedie's (2000) trim-and-fill method, which considerably increased the effect sizes (ORs ranged from 3.54 to 5.28; see Table 3). These results suggest that publication bias may not be an actual concern (Murad *et al*., 2018).

**Table 3.** Results of meta-analyses of the association between forms of childhood maltreatment and suicide attempts in prisoners

			Effect size analyses			Heterogeneity	Publication bias	
	Total *k*	Total *N*	OR	95% CI	*p* value	*I*^2^	Egger's *p*	Trim-and-fill OR (95% CI)
Suicide attempts								
Sexual abuse	16^a^	9835	2.68	1.86–3.86	<0.001	92.4%	0.001	3.92 (1.50–10.26)
Physical abuse	11^b^	5572	2.16	1.60–2.91	<0.001	86.6%	0.001	3.66 (1.39–9.60)
Emotional abuse	9	5258	2.70	1.92–3.79	<0.001	74.0%	0.001	5.28 (1.02–27.8)
Emotional neglect	7^b^	3123	2.29	1.69–3.10	<0.001	41.9%	–	–
Physical neglect	7^b^	3049	1.57	1.27–1.94	<0.001	89.2%	–	–
Combined abuse	13^b^	10 219	3.09	2.14–4.45	<0.001	91.9%	0.001	4.48 (1.33–15.13)

*k*, number of independent effect sizes; OR, pooled odds ratio; *N*, number of participants.

a Two outliers were dropped from the analyses.

b One outlier was dropped from the analyses.

### Meta-regressions examining the impact of methodological variations on the associations between distinct forms of childhood maltreatment and suicide attempts in prisoners

The number of pooled studies only allowed meta-regression analyses to be conducted for the associations between sexual, physical, emotional and combined abuse, and suicide attempts. The results of the univariate meta-regression analyses revealed that participant variables, including mean age (*p* values ranged between 0.61 and 0.87) and percentage of males (*p* values ranged between 0.34 and 0.97), and methodological variations, such as screening measures for childhood maltreatment (*p* values ranged between 0.44 and 0.86), screening measures for suicide attempts (*p* values ranged between 0.73 and 0.96), timeframe of suicide attempts (*p* values ranged between 0.40 and 0.79) and critical appraisal score (*p* values ranged between 0.31 and 0.87), across studies did not affect the strength of the associations between different forms of abuse and suicide attempts (see online Supplementary Appendix D for the results of the meta-regression analyses).

## Discussion

This is the first systematic meta-analytic review to examine the relationship between core forms of childhood maltreatment and suicide attempts in prisoners. We reported results from a total of 16 586 prisoners. The rates of childhood maltreatment experiences ranged from 29% to 68%, whereas the rate of suicide attempts in prisoners was 23%. There were two key findings. First, we demonstrated that suicide attempts were strongly linked, cross-sectionally, with experiences of childhood abuse and/or neglect in prisoners, which remained unaffected by methodological variations. In particular, prisoners who reported experiences of combined abuse/neglect, sexual and emotional abuse were three times more likely to engage in suicide attempts compared to those prisoners who had not experienced such events. Physical abuse and emotional neglect were associated with twice the likelihood for suicide attempts. Finally, prisoners who reported being physically neglected during childhood were found to have 1.5 more suicide attempts in adulthood compared with prisoners who had not experienced abuse or neglect. It must be highlighted that these results reflect cross-sectional rather than longitudinal data. Consequently, neither temporal precedence nor causation can be demonstrated.

To date there have been limited attempts to link the strong relationship between forms of childhood maltreatment and suicidal experiences, with contemporary models of suicidal thoughts and behaviors (Johnson *et al*., 2008; O'Connor and Nock, 2014; Klonsky and May, 2015; O'Connor and Kirtley, 2018; O'Connor and Portzky, 2018). Four key priorities would advance this literature. First, researchers should identify socio-economic and epidemiological factors which are associated with a high prevalence of suicidal thoughts, acts, and fatalities in vulnerable prisoners (Rivlin *et al*., 2013; Fazel *et al*., 2017). Second, a better understanding of the ways in which different forms of childhood abuse may exacerbate symptoms of mental health problems is needed. Examples of such symptoms include avoidance, hyperarousal, and command hallucinations which may trigger and/or amplify suicidal thoughts and behaviors (Panagioti *et al*., 2017). Third, future research should examine the ways in which experiences of childhood abuse feed into more transdiagnostic approaches which seek to better understand the psychological pathways leading to suicidal thoughts, urges, plans, acts and deaths (Bolton *et al*., 2007; Johnson *et al*., 2008; O'Connor and Kirtley, 2018). The fourth issue follows in that there should be an endeavor to investigate how symptom-specific contributors to suicidal experiences interact with putative psychological suicide mechanisms (Gooding *et al*., 2015). Many of the contemporary models of suicide highlight that important mediators in the relationship between childhood maltreatment and suicide attempts, which include perceptions of being socially isolated, feeling a burden, being overwhelmed by, for example, strong labile emotions, feeling defeated and/or trapped, and having no hope for the future, are central to the pathways to suicidal experiences (Johnson *et al*., 2008; Van Orden *et al*., 2010; Palmier-Claus *et al*., 2012; O'Connor and Kirtley, 2018). Therefore, it is paramount that future studies explore the interactions between past and current traumatic experiences in prisoners that may amplify these types of feelings and perceptions using qualitative and quantitative longitudinal and micro-longitudinal designs. Finally, a key component to consider is the development and activation of suicide schema in people who have experienced child abuse (Lau *et al*., 2004). Suicide schemas are under-researched but have been found to be extensive in people with non-affective psychosis and also post-traumatic stress disorder (Tarrier *et al*., 2007; Pratt *et al*., 2010; Panagioti *et al*., 2015). It is important to determine how suicide schema develop and become more extensive over time as a result of current and past childhood abuse and/or neglect, especially in those vulnerable to suicidal experiences (i.e. prisoners).

None of the identified studies examined the impact of potentially important moderators on the relationship between childhood maltreatment and suicide attempts. Examples might include such variables as length of offense, life events, such as homelessness or running away from home as an adolescent (Kenny *et al.*, 2008; Rivlin *et al*., 2013; Bhatta *et al*., 2014; Sánchez *et al*., 2019), and adverse prison-related experiences, including social isolation and victimization by other prisoners (Blaauw *et al*., 2002; Suto and Arnaut, 2010). Perhaps most importantly, we should prioritize studies examining key factors that attenuate or buffer the relationship between childhood abuse/neglect and suicide attempts. For example, Rivlin *et al*. (2011) suggested that an increased level of care from professionals or prison staff, especially when prisoners engage in precursor non-suicidal self-harm behaviors, may be effective at reversing such outcomes. However, while examination of mediators and/or moderators is important, studies have rarely been able to examine these types of mediators and moderators in prisoners who have attempted or died by suicide, because converging data on pertinent and meaningful mediators and/or moderators together with suicide attempts and fatalities are rare. Clearly, future research should further explore the significance of these factors on suicidal experiences in prisoners.

Second, we identified a paucity of studies exploring the link between childhood abuse/neglect and the different modes of suicide behavior other than attempts, including suicidal ideation, urges, and plans. We only identified four studies exploring the link between any form of childhood maltreatment and suicidal ideation or suicide risk (Blaauw *et al*., 2002; Zhang *et al*., 2010; Sergentanis *et al*., 2014; Boonmann *et al*., 2016). This is an important gap in the literature because there is compelling evidence suggesting that among those individuals experiencing lifetime suicidal ideation with or without plans the likelihood of acting upon their thoughts ranges between 30% and 55% (Nock *et al*., 2008). Furthermore, alleviating the immense psychological distress associated with having suicidal thoughts, urges and/or forming suicide plans is an important clinical target (Tarrier *et al*., 2013).

Recent evidence suggests that cognitive-behavioral suicide prevention therapies can be successfully extended within prison settings. For example, Pratt *et al*. (2015) found that this treatment approach was promising in reducing suicide risk in male prisoners considered at high risk for suicide. Given preliminary evidence suggesting that the format of cognitive-behavioral therapy (e.g. individual *v.* group) does not alter its effectiveness in reducing psychological symptoms such as depression and anxiety in prisoners (Khodayarifard *et al*., 2010), which are strongly linked to suicidal acts, research on scaling up clinical therapies for groups of high-risk prisoners is essential.

It is also worth noting that more than half of the included studies were undertaken in the USA, followed by Italy and Australia. This is not a surprising, given that the USA has among the highest rates of incarceration globally (U.S. Department of Justice, 2016). However, the USA is among the countries with the lowest rates of prison suicide (Fazel *et al*., 2017). Countries such as England, France, Norway and Sweden, where incarceration rates are lower, contributed a single study (per country) to the analysis. Interestingly, these countries have the highest prison suicide death rates worldwide. Differences in definitions of suicide deaths in prisoners, which was initially suspected to account for this discrepancy, did not affect overall prison suicide rates. It is, therefore, important that more research is conducted to identify key factors that contribute to these outcomes, including country specific provision of mental health care, amount of daily activity, and quality of staff-prisoner relationships, among others.

There are five main limitations of the current study which warrant discussion. First, heterogeneity across the majority of the comparisons undertaken was moderate to high, which is, perhaps, to be expected. To address this, we applied random effect models to adjust for between-study variations. Second, we found an indication of publication bias for the majority of the comparisons examined. To attenuate this bias, we employed the fill-and-trim approach which increased the effect sizes. Although these results suggest that the strong link between experiences of childhood maltreatment and suicide attempts in prisoners may remain unaffected (e.g. Murad *et al*., 2018), they should still be interpreted with caution. Third, the number of studies included in some comparisons was small. As such, these findings may warrant further investigation. Fourth, this review focused exclusively on core types of childhood maltreatment and, as such, particular types of adverse childhood events (e.g. parental deaths and divorces) were not considered. Future research should explore these relationships in more depth. Fifth, although it would have been more informative to differentiate between the prevalence of lifetime suicide attempts and those attempts that occurred while in the prison system, the lack of such information from the included studies prevented this distinction from being made. However, it is acknowledged that the most helpful information for correctional staff would be rates of suicide attempts while in prison separated from those which preceded incarceration or followed release from prison. That said, it is important to note that many prisoners return to prison after release, making the dynamic between factors which precipitate suicide attempts in prison and those which precipitate suicide attempts after release difficult to capture. In this regard, qualitative work which probes the triggers for suicidal thoughts, plans and attempts while in prison and after release, and the interactions between the effects of such different contexts with child abuse, may be of optimal practical help going forward. With these caveats acknowledged, a clear message from this meta-analysis is that prisoners who have experienced child abuse and/or neglect may be the most vulnerable with respect to having different forms of suicidal thoughts, plans and acts.

This study has three noteworthy strengths. First, it is the first systematic review with meta-analysis which incorporated an extensive synthesis of the association between core forms of childhood maltreatment and suicide attempts in prisoners confirming their robust associations with suicide attempts. Second, the current meta-analysis was conducted in compliance with PRISMA and MOOSE guidelines. Third, the methodological quality of the included studies was critically evaluated and rated.

## Conclusions

In summary, this is the first systematic meta-analytic review of the relationship between childhood maltreatment and suicide attempts that was conducted in prisoners. Although this relationship has yet to be fully explored, we demonstrated that a strong link exists between forms of childhood maltreatment and suicide attempts. No evidence exists regarding other modes of suicide behaviors, including suicidal ideation, suicide plans and deaths by suicide. We recommend that further research employ more robust, higher quality designs, such as, longitudinal, micro-longitudinal, qualitative and/or mixed designs, to corroborate and expand the findings of the current review. Taken together, these findings highlight, as a matter of urgency, the need for targeted suicide prevention priorities for prisoners with a focus on ameliorating the effects of childhood maltreatment on suicidal thoughts, behaviors and attempts (House of Commons Library, 2018). These initiatives should be a priority at the levels of policy making and institutional reform, whereby the provision of different modes of suicide focused psychological therapy targeting childhood experiences of maltreatment (Pratt *et al*., 2015) is conducted in tandem with robust staff awareness and training programs (Joe *et al*., 2017).

## Supplementary material

For supplementary material accompanying this paper visit https://doi.org/10.1017/S0033291719002848. click here to view supplementary material
