# Supplementary material for: Childhood maltreatment and suicide attempts in prisoners: a systematic meta-analytic review
Source: Psychol Med. 2019 Oct 30;50(1):1–10. doi: 10.1017/S0033291719002848 (PMC6945324; doi:10.1017/S0033291719002848)
Supplement: Supplementary file 1 [file S0033291719002848sup.zip › S0033291719002848sup001.docx]

B

A

C

D

*Appendix B:* Funnel plots for effect sizes.

Childhood maltreatment and suicide attempts: (A) sexual abuse, (B) physical abuse, (C) emotional abuse, and (D) childhood maltreatment.
