# Supplementary material for: Childhood maltreatment and suicide attempts in prisoners: a systematic meta-analytic review
Source: Psychol Med. 2019 Oct 30;50(1):1–10. doi: 10.1017/S0033291719002848 (PMC6945324; doi:10.1017/S0033291719002848)
Supplement: Supplementary file 1 [file S0033291719002848sup.zip › S0033291719002848sup003.docx]

Appendix D

*Figure D1. Forest plot of the rates of suicide attempts in prisoners. Note random effects model used.*

*Figure D2. Forest plot of the percentage of prisoners who reported experiences of sexual abuse. Note random effects model used.*

*Figure D3. Forest plot of the percentage of prisoners who reported experiences of physical abuse. Note random effects model used.*

*Figure D4. Forest plot of the percentage of prisoners who reported experiences of emotional abuse. Note random effects model used.*

*Figure D5. Forest plot of the percentage of prisoners who reported experiences of emotional neglect. Note random effects model used.*
