# Supplementary material for: Childhood maltreatment and suicide attempts in prisoners: a systematic meta-analytic review
Source: Psychol Med. 2019 Oct 30;50(1):1–10. doi: 10.1017/S0033291719002848 (PMC6945324; doi:10.1017/S0033291719002848)
Supplement: Supplementary file 1 [file S0033291719002848sup.zip › S0033291719002848sup005.docx]

Figure 2

*Figure 2.1. Forest plot of the association between sexual abuse and suicide attempts. Note random effects model used. OR = Odds ratio.*

*Figure 2.2. Forest plot of the association between physical abuse and suicide attempts. Note random effects model used. OR = Odds ratio.*

*Figure 2.3. Forest plot of the association between emotional abuse and suicide attempts. Note random effects model used. OR = Odds ratio.*

*Figure 2.4. Forest plot of the association between emotional neglect and suicide attempts. Note random effects model used. OR = Odds ratio.*

*Figure 2.5. Forest plot of the association between physical neglect and suicide attempts. Note random effects model used. OR = Odds ratio.*

*Figure 2.6. Forest plot of the association between combined abuse and suicide attempts. Note random effects model used. OR = Odds ratio.*
