# Supplementary material for: Childhood maltreatment and suicide attempts in prisoners: a systematic meta-analytic review
Source: Psychol Med. 2019 Oct 30;50(1):1–10. doi: 10.1017/S0033291719002848 (PMC6945324; doi:10.1017/S0033291719002848)
Supplement: Supplementary file 1 [file S0033291719002848sup.zip › S0033291719002848sup004.docx]

Appendix C

*Meta-regression analyses*

|  | Univariate Meta-regression Analyses | | | | | | |  |  |  |
| --- | --- | --- | --- | --- | --- | --- | --- | --- | --- | --- |
|  | *b* | *SE* | | | *P* value | | | |  |  |
| **Suicide attempts** |  | |  | | |  | | | |  |
| Sexual abuse |  | | |  | | |  | | | |
| Mean age | -0.02 | | | 0.14 | | | 0.87 | | | |
| Male gender % | 0.14 | | | 1.15 | | | 0.90 | | | |
| CM measure (Interview/scale) | 0.23 | | | 1.29 | | | 0.86 | | | |
| Suicidality measure (Interview/scale) | -0.33 | | | 1.27 | | | 0.80 | | | |
| Timeframe of attempts (lifetime/current/both) | -0.25 | | | 0.51 | | | 0.63 | | | |
| Critical appraisal score (Low/high) | 0.18 | | | 1.05 | | | 0.87 | | | |
| Physical abuse |  | | |  | | |  | | | |
| Mean age | 0.05 | | | 0.13 | | | 0.68 | | | |
| Male gender % | 0.09 | | | 1.02 | | | 0.93 | | | |
| CM measure (Interview/scale) | 0.30 | | | 1.10 | | | 0.79 | | | |
| Suicidality measure (Interview/scale) | -0.75 | | | 2.09 | | | 0.73 | | | |
| Timeframe of attempts (lifetime/current/both) | 0.15 | | | 0.55 | | | 0.79 | | | |
| Critical appraisal score (Low/high) | -1.32 | | | 1.24 | | | 0.31 | | | |
| Emotional abuse |  | | |  | | |  | | | |
| Mean age | 0.07 | | | 0.21 | | | 0.75 | | | |
| Male gender % | -0.27 | | | 6.16 | | | 0.97 | | | |
| CM measure (Interview/scale) | -1.06 | | | 1.83 | | | 0.58 | | | |
| Suicidality measure (Interview/scale) | -0.24 | | | 2.87 | | | 0.94 | | | |
| Timeframe of attempts (lifetime/current/both) | -0.60 | | | 1.05 | | | 0.59 | | | |
| Critical appraisal score (Low/high) | -0.96 | | | 1.87 | | | 0.62 | | | |
| Combined abuse |  | | |  | | |  | | | |
| Mean age | 0.11 | | | 0.21 | | | 0.61 | | | |
| Male gender % | 1.40 | | | 1.40 | | | 0.34 | | | |
| CM measure (Interview/scale) | -1.13 | | | 1.42 | | | 0.44 | | | |
| Suicidality measure (Interview/scale) | 0.11 | | | 2.30 | | | 0.96 | | | |
| Timeframe of attempts (lifetime/current/both) | 0.92 | | | 1.04 | | | 0.40 | | | |
| Critical appraisal score (Low/high) | -1.25 | | | 1.45 | | | 0.41 | | | |

*Note. CM* = Childhood maltreatment.
